# Soluble CD40 Ligand in Sera of Subjects Exposed to *Leishmania infantum* Infection Reduces the Parasite Load in Macrophages

**DOI:** 10.1371/journal.pone.0141265

**Published:** 2015-10-21

**Authors:** Fabrícia Alvisi de Oliveira, Aline Silva Barreto, Lays G. S. Bomfim, Talita Rebeca S. Leite, Priscila Lima dos Santos, Roque Pacheco de Almeida, Ângela Maria da Silva, Malcolm S. Duthie, Steven G. Reed, Tatiana Rodrigues de Moura, Amélia Ribeiro de Jesus

**Affiliations:** 1 Laboratório de Biologia Molecular, Hospital Universitário, Universidade Federal de Sergipe, Aracaju, Brazil; 2 Instituto de Investigação em Imunologia, São Paulo, Brazil; 3 Infectious Disease Research Institute (IDRI), Seattle, Washington, United States of America; Louisiana State University, UNITED STATES

## Abstract

**Background:**

While CD40L is typically a membrane glycoprotein expressed on activated T cells and platelets that binds and activates CD40 on the surface on antigen presenting cells, a soluble derivative (sCD40L) that appears to retain its biological activity after cleavage from cell membrane also exists. We recently reported that sCD40L is associated with clinical resolution of visceral leishmaniasis and protection against the disease. In the present study we investigated if this sCD40L is functional and exerts anti-parasitic effect in *L*. *infantum*-infected macrophages.

**Methodology/Principal Findings:**

Macrophages from normal human donors were infected with *L*. *infantum* promastigotes and incubated with either sera from subjects exposed to *L*. *infantum* infection, monoclonal antibodies against human CD40L, or an isotype control antibody. We then evaluated infection by counting the number of infected cells and the number of parasites in each cell. We also measured a variety of immune modulatory cytokines in these macrophage culture supernatants by Luminex assay. The addition of sCD40L, either recombinant or from infected individuals’ serum, decreased both the number of infected macrophages and number of intracellular parasites. Moreover, this treatment increased the production of IL-12, IL-23, IL-27, IL-15, and IL1β such that negative correlations between the levels of these cytokines with both the infection ratio and number of intracellular parasites were observed.

**Conclusions/Significance:**

sCD40L from sera of subjects exposed to *L*. *infantum* is functional and improves both the control of parasite and production of inflamatory cytokines of infected macrophages. Although the mechanisms involved in parasite killing are still unclear and require further exploration, these findings indicate a protective role of sCD40L in visceral leishmaniasis.

## Introduction

Visceral leishmaniasis (VL) is a chronic systemic disease caused by infection with the protozoan parasite *Leishmania infantum (chagasi)*. VL patients present with an intense parasitization of the spleen, liver and bone marrow, followed by symptoms that include fever, hepatosplenomegaly, anemia, leucopenia, and severe weight loss. VL is frequently fatal if not treated.

Control of leishmania infection is mediated by macrophages and is associated with production of inflammatory cytokines, such as IL-1β, TNF-α, IL-6 and IL-12 family members [1–5]. These cytokines stimulate microbicidal mechanisms, such as nitric oxide production, that are essential to parasite killing and clearance in experimental models, although in humans this is still controversial [1,5–7]. IL-12 also drives the differentiation of antigen-specific CD4+ T cells into IFN-γ and TNF producing Th1 cells which are critically required for protection [8], [9]. Conversely, establishment of *Leishmania* infection is associated with an impairment of specific Th1 responses to leishmania antigens [10] and high levels of IL-10 [11–13] that deactivates various signaling pathways [14] required for effective immune responses against the parasite.

The interaction of CD40 with its ligand CD40L represents an important costimulatory pathway required for the generation of effective T cell responses [15,16]. CD40 is present on surface on antigen presenting cells (APCs) such as B cells, monocytes, macrophages and dendritic cells, as well as on the membrane of various non-immune cells, such as endothelial and epithelial cells [16]. CD40L is primarily expressed on activated CD4^+^ T cells, but is also present on platelets and a small proportion of CD8^+^ T cells [16]. Stimulation through CD40 enhances the survival of APCs and promotes the secretion of IL-1, IL-6 IL-8, IL-10, IL-12, TNF-α, MIP-1α and enzymes such as matrix metalloproteinases, as well as synthesis of NO [17–20]. In numerous infectious diseases, the interaction of CD40 and CD40L can determine resistance or susceptibility to infection [21–23]. The role of costimulatory importance of CD40-CD40L signaling is well demonstrated in experimental models of leishmaniasis, [24–28], with strong CD40-CD40L signaling inducing IL-12 production by macrophages whereas weak signaling induces IL-10 production [29].

CD40L is also found as a soluble derivative (sCD40L) that is cleaved from activated T cells that appears to retain the ability to bind and activate CD40 on APC [30,31]. Some studies in cardiovascular disease and sepsis have described enhanced levels of sCD40L as an inflammatory mediator, and the presence of sCD40L is considered as a risk factor, and as an indicator of poor outcome, for these diseases [32,33]. However, we recently reported that sCD40L is associated with clinical resolution of VL. A gradual increase in the levels of serum sCD40L was observed during treatment and levels were negatively correlated with spleen size and parasite load. We also observed high levels of sCD40L in non-diseased individuals living in VL-endemic regions, suggesting that sCD40L may contribute to protection [34]. In the present study, we demonstrate that sCD40L in the serum of individuals exposed to *L*. *infantum* infection can bind to CD40 on *L*. *infantum*-infected macrophages and that it helps to control the infection.

## Methods

### Activity of serum sCD40L in *L*. *infantum* infected macrophages

Macrophages were derived from peripheral blood mononuclear cells (PBMC) isolated from the blood of healthy donors. Briefly, heparinized venous blood was obtained and PBMC separated by Ficoll Hypaque gradient (Sigma Aldrich). The cells were washed twice, counted and ressuspended in RPMI 1640 (Sigma Chemical) supplemented with 10% FBS and 1% penicillin then seeded in eight chamber Lab-Tek glass tissue culture slide (Nalge Nunc International) at 3x10^5^ cells/well in a volume of 0.2 ml. After the cells were allowed to adhere for 2 h at 37°C in 5% CO_2_, non-adherent cells were removed by extensive washing. The adherent monocytes were incubated in supplemented medium at 37°C, 5% CO_2_ for 7 days to allow them to differentiate into macrophages. Macrophages were subsequently infected by adding stationary-phase *L*. *infantum* promastigotes (MHOM/BR/2009/LVHSE17) at a parasite to macrophage ratio of 10:1. Extracellular parasites were removed 2 hours later by extensive washing, and the infected cells incubated for a further 72 h.

**Fig 1:**
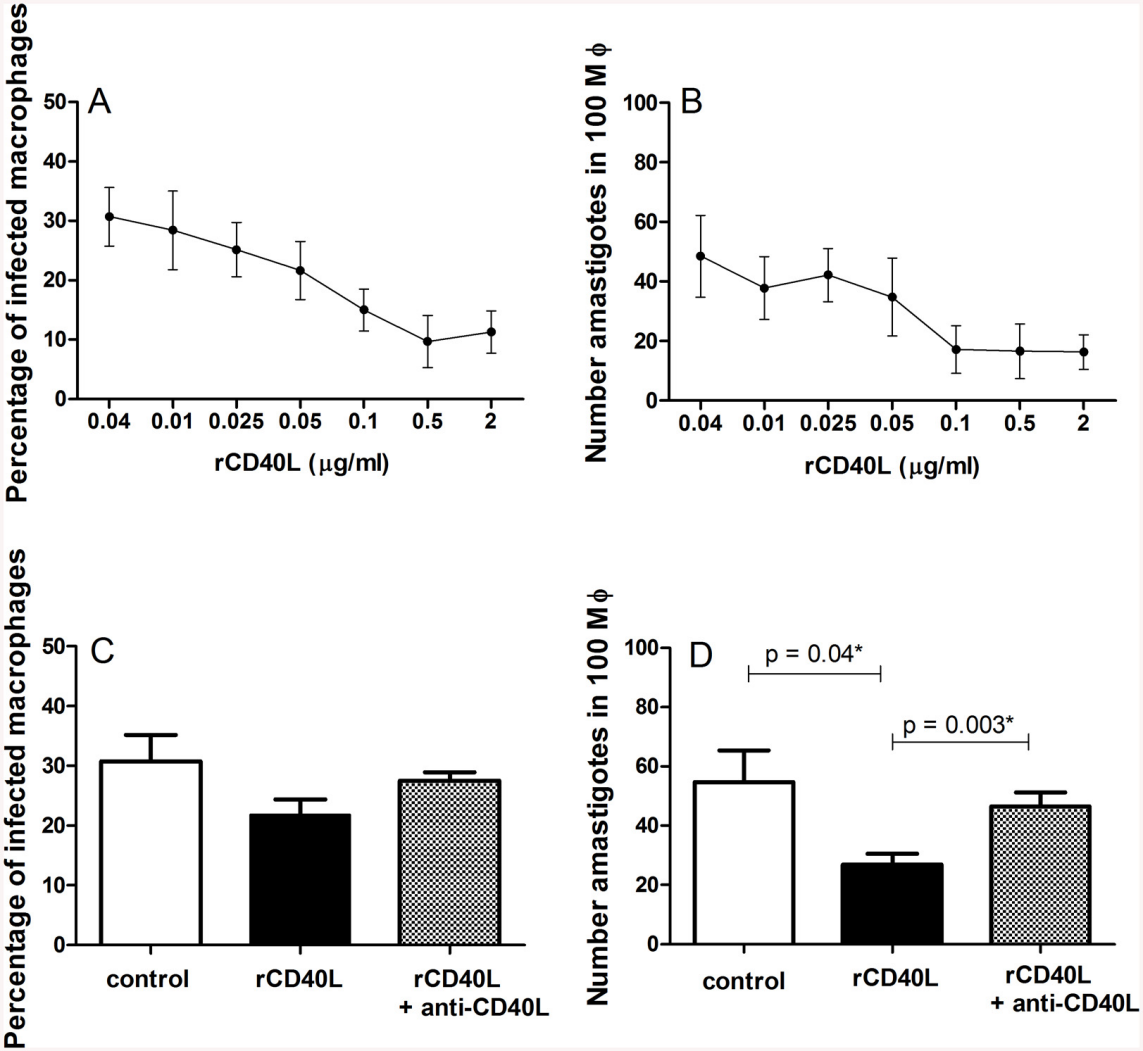
Treatment with recombinant CD40L (rCD40L) reduces *L*. *infantum* infection in macrophages. *L*. *infantum* infection levels in macrophages were determined after 72 h incubation in complete RPMI media (control) or in the presence of rCD40L. A dose-response curve was evaluated across several concentrations of rCD40L, with (A) the number of infected macrophages/100 macrophages and (B) the number of amastigotes/100 macrophages determined. Secondly, rCD40L at 2 μg/ml, either alone or in combination with anti-CD40L (10 μg/ml) were added to the macrophage cultures. In (C) the number of infected macrophages/100 macrophages and in (D) the number of amastigotes/100 macrophages were measured. The results represent the mean and SD of 4 donors, with each experiment performed in duplicate. *Mann Whitney test.

To examine the role of sCD40L, infected cells were incubated with 20% human serum or 10 μg/ml monoclonal antibodies against human CD40L or an isotype control (both R&D Systems, Minneapolis, MN). The sera used were selected from DTH positive subjects and cured VL patients, with no signs of infection, with high levels of sCD40L (ranging from 30,618 to 145,551 pg/ml). Based on previous report [35], recombinant human CD40L (R&D Systems) was added at 2 μg/ml for both 2 h and 72 h incubation after infection. This concentration was also selected as being fully saturating as demonstrated in a dose-response curve (Fig 1). The supernatants were collected and stored at—80°C until cytokine measurement. To assess the infection ratio, cells were stained with Panotico fast (LaborClin, PR, BRA). The infection ratio was measured by counting the number of infected cells/100 macrophages and the number of parasites/100 macrophages, each of which was conducted in a blinded fashion and by three independent observers. All conditions were performed in duplicate. Ethical approval was obtained from the Hospital Universitário from Universidade Federal de Sergipe, Comissão Nacional de Ética em Pesquisa (CONEP), CAAE 0151.0.107.000–07 and CAAE 0123.0.107.000–11. All cured VL patients and normal donors signed an informed consent.

### Cytokine measurements

Concentrations of IL-1β, IL-6, IL-12p70, IL-15, IL-23, IL-27, and TNF-α in the culture supernatants of infected macrophages were determined by Luminex assay, according to the manufacturer’s instructions (Millipore, Massachusetts, USA).

### Statistical analysis

D’Agostino-Pearson normality test was applied. Mann Whitney test was used for two groups comparisons. Paired analysis was performed by Wilcoxon signed rank test for non-Gaussian data or paired T test in data that fit Gaussian distribution, for comparison of cytokine levels between the experiments containing the sCD40L serum plus anti-CD40L or isotype control antibodies. Correlations between infection levels and cytokines released in culture were performed by Spearman or Pearson tests, according to the results of the normality test. All tests were performed using GraphPad Prism, version 5.03 (GraphPad Software, San Diego, USA). An α < 5% (p < 0.05) was considered statistically significant.

## Results

### Recombinant CD40L can control the parasite load of *L*. *infantum*-infected macrophages

Our previous report identified an inverse relationship between sCD40L levels in the sera of VL patients and cure in response to treatment, implicating a role for sCD40L in protection against *L*. *infantum*. To determine if sCD40L could have a protective role against *L*. *infantum* infection, we added recombinant human CD40L (rCD40L) in combination with anti-CD40L to human macrophages infected with *L*. *infantum*. Firstly, we demonstrated that a wide range of rCD40L concentrations could reduce the infection (Fig 1A and 1B) and determined to use 2 μg/ml of rCD40L in further experiments. Treatments did not interfere with parasite uptake by the macrophages (data not shown) and infection levels after 2 hours of incubation were comparable between the control without any stimulus (63.7% ± 12.61) and treatment with either rCD40L alone (74.3% ± 6.94) or treatment with rCD40L and anti CD40L together (66.0% ± 6.21). After 72 h of incubation, however, the presence of human rCD40L caused a significant decrease of the number of amastigotes per macrophages relative to control cells, and this could be reversed by the addition of anti-CD40L (Fig 1C and 1D). These data demonstrate that sCD40L has the potential to alter parasite growth in infected macrophages.

### sCD40L in the sera of *L*. *infantum* infected individuals limits parasite growth in macrophages

**Fig 2:**
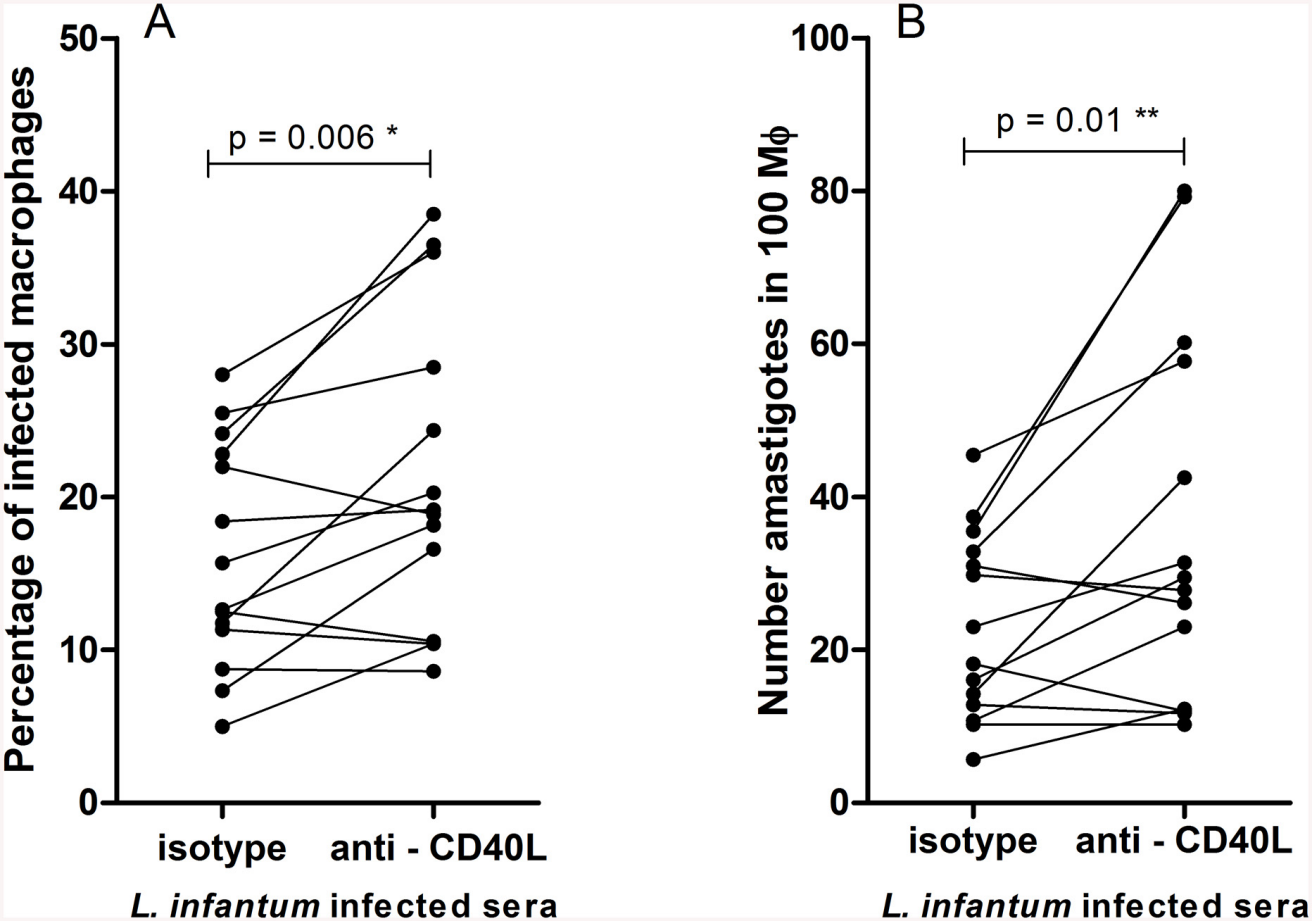
Serum sCD40L improves the killing of *L*. *infantum* by infected macrophages. *L*. *infantum* infection levels in macrophages were assessed after 72 h incubation with 20% serum from subjects infected with *L*. *infantum* in the presence of isotype control antibody (10 μg/ml) or anti-human CD40L (10 μg/ml). The (A) number of infected macrophages/100 macrophages and; (B) number of amastigotes/100 macrophages are shown. n = 14, *Paired t test and ** Wilcoxon signed rank test.

To determine if sCD40L in the serum of patients could afford some protection against *L*. *infantum*, we added serum from infected individuals to parasite infected macrophages *in vitro*. In the majority of cases the blockade of sCD40L increased both the number of infected macrophages and the number of parasites/ 100 macrophages over levels observed in macrophages incubated with control antibody (10 of 14 paired cultures in each case; Fig 2). In each case, the infection levels were increased by over fifty percent by anti-CD40L treatment. These data indicate that sCD40L in the sera of VL patients is functional and limits the ability of *L*. *infantum* to grow in macrophages.

### sCD40L enhances inflammatory cytokine production by *L*. *infantum* infected macrophages

**Fig 3:**
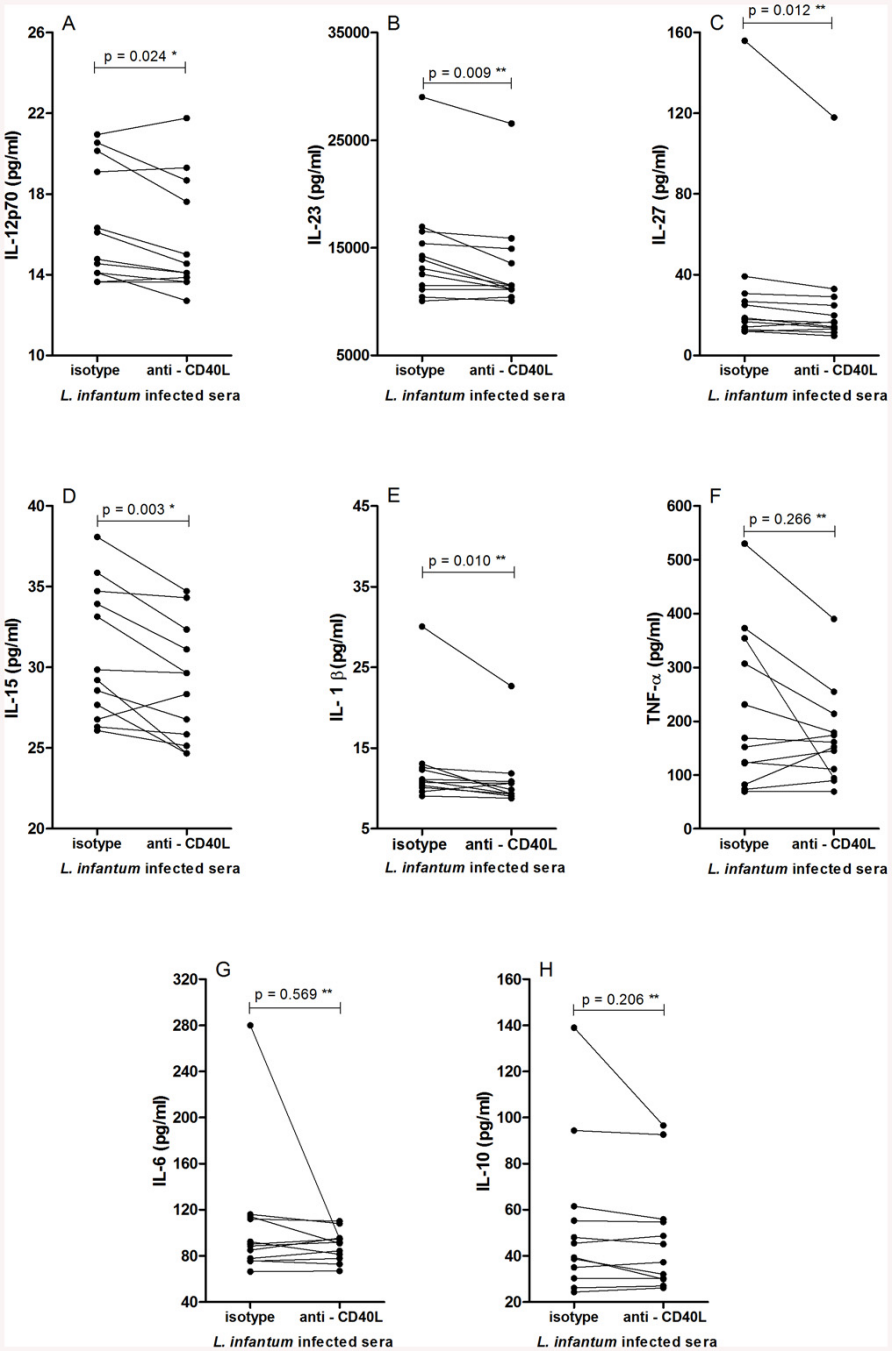
Blockade of serum sCD40L reduces inflammatory cytokine production by infected macrophages. Levels of cytokines in culture supernatant of macrophages infected with *L*. *infantum* were measured after 72 hr incubation with 20% serum from subjects infected with *L*. *infantum* in presence of isotype control antibody (10 μg/ml) or anti human CD40L (10 μg/ml) (A) IL-12; (B) IL-23; (C) IL-27; (D) IL-15, E (IL-1β), F (TNF-α). G (IL-6) and H (IL-10). n = 12, *Paired t test, ** Wilcoxon signed rank test.

**Fig 4:**
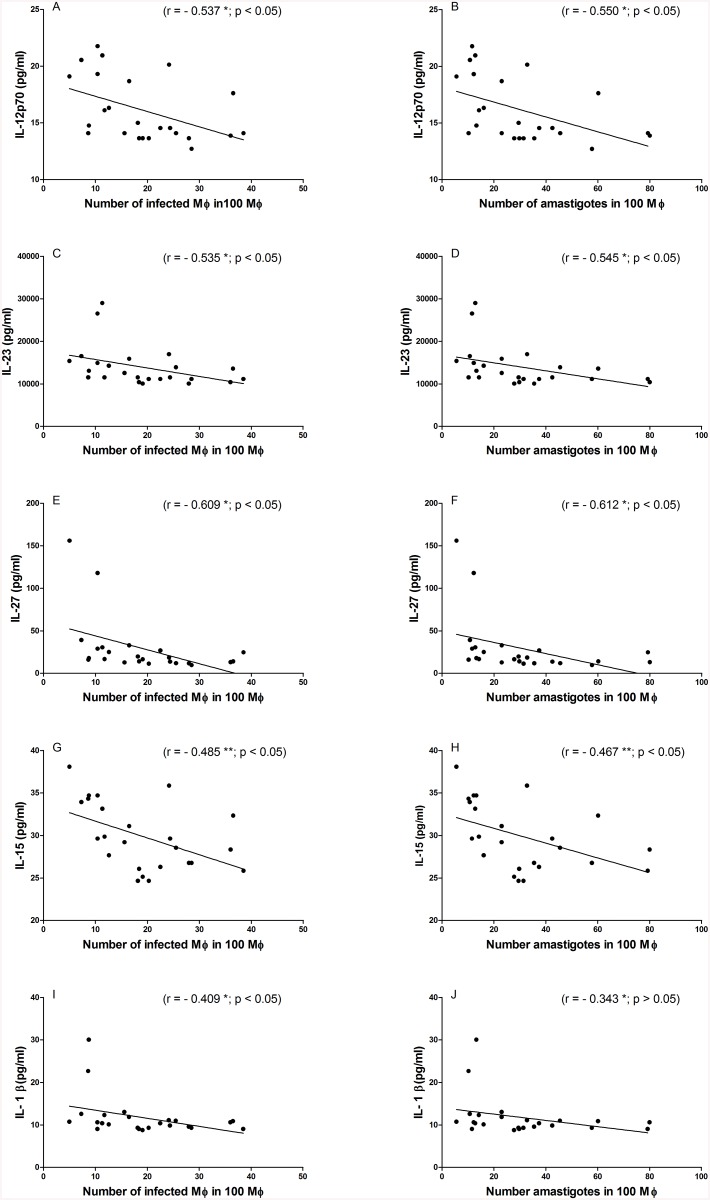
Cytokine levels in supernatants of *L*. *infantum* infected macrophages negatively correlate with infection levels. Correlation between cytokine levels in culture supernatants incubated with serum sCD40L (n = 12) and with sCD40L plus anti-CD40L (n = 12) with the number of infected macrophages/100 macrophages and the number of amastigotes/100 macrophages from these experiments. (A) and (B) IL-12; (C) and (D) IL-23; (E) and (F) IL-27; (G) and (H) IL-15; (I) and (J) IL-1β; n = 24. *Spearman correlation test and ** Pearson test.

CD40L is known to influence various immune modulatory mechanisms, among them cytokine secretion. We therefore also evaluated how the addition of serum from *L*. *infantum*-infected individuals impacted the production of inflammatory and regulatory cytokines by infected macrophages. Incubations were conducted in the presence or absence of anti-human CD40L. The production of IL-12, IL-15, IL-23, IL-27 and IL1β were all significantly reduced by the blockade of sCD40L (Fig 3). In contrast, the production IL-6, IL-10 and TNF was not adversely affected (Fig 3). Interestingly, further examination revealed negative correlations between the levels of IL-12p70, IL-15, IL-23 and IL-27 measured in the culture supernatants and both the number of infected macrophages and the number of amastigotes (Fig 4). IL-1β levels correlated with the number of amastigotes (Fig 4). Thus, the blockade of sCD40L in the serum of infected individuals reduced cytokine production, implying that sCD40L retains its immune regulatory functions during *L*. *infantum* infection.

## Discussion

We previously reported that higher levels of sCD40L are associated with clinical resolution of VL [34]. In this study, we demonstrate that sCD40L in the serum of infected subjects is active and can potentiate both the ability to control the infection, and inflamatory cytokine production, of *L*. *infantum*-infected macrophages. Although other molecules present in the sera may also have an effect, our demonstration that sCD40L neutralization by specific antibodies confirms the specificity of the effect of sCD40L on parasite killing.

CD40L is typically present in cell membrane, mainly of activated T cells. It has been demonstrated that stimulation through CD40 activates tumor necrosis factor receptor-associated factors (TRAF 1, 2, 3, 5 and 6), which in turn stimulate various kinases (p38 MAPK, ERKs, pI3K) and induce NF-κβ- and STAT-dependent gene expression. Together, this results in the production of antibodies, enzymes and a variety of cytokines including IL-1β, IL-6, IL-8, IL-10, IL-12 and TNF-α [16,36,37]. Additionally, CD40/CD40L signaling on monocytes/macrophages induces the production of NO [17,38], a major host defense mechanism against *Leishmania* [7]. The important role of CD40-CD40L signaling for protection in leishmaniasis has been demonstrated using recombinant proteins and knockout mice [25,27,28,39]. Furthermore, the role of CD40L in acquired resistance to leishmania infection is supported by the ability of this molecule to potentiate vaccine-induced immunity against *L*. *major* infection [40,41]. Despite the broad functional knowledge regarding the importance of the membrane form of CD40L, the role of sCD40L remains unclear. However, by demonstrating that sCD40L levels progressively increase during clinical resolution of the disease and that levels negatively correlate with parasite burden, our previous study presented circumstantial evidence of a protective role in VL for sCD40L [34]. Although sCD40L is a cleaved/shed form, it retains the ability to bind CD40 and activate macrophages, independently of T cells [31]. sCD40L has been described as a mediator of inflammation and a marker of poor prognosis in arterial coronary disease [32,42,43]. High sCD40L levels are detected in the serum of HIV-1 and sepsis patients, and are associated with poor prognosis in both diseases [33,44]. Considering that VL patients have impaired antigen-specific T cell responses during active disease [10], sCD40L could represent an important T cell independent regulator of macrophage function in VL. Indeed, our results demonstrate that the presence of sCD40L in serum helps to control infection levels, most likely by potentiating the microbicidal mechanisms of infected macrophages.

Our data also indicates that serum sCD40L increases the production of various cytokines (IL-12p70, IL-23, IL-27, IL-15 and IL-1β) by infected macrophages *in vitro*. Interestingly, these cytokines were negatively correlated with the number of infected macrophages. Several publications have demonstrated that CD40-CD40L signaling increases the production of IL-12, IL-23, IL-27 and IL-1β [45–48], which play a role on the activation of macrophage microbicidal activities [49–52]. *L*. *major* amastigotes can modulate the signaling pathway downstream of membrane CD40 engagement by inducing ERK1/2 and IL-10 production, which inhibits the p38MAPK/IL12 pathway resulting in replication of parasite and persistence of infection [35]. This down regulatory response can be overcome by stronger CD40-sCD40L signaling. Thus, the presence of sCD40L could be important for restoration of IL-12 production. Furthermore, the presence of IL-12 is crucial to induce a Th1 phenotype [53,54] during antigen presentation, required for an effective immune response in visceral leishmaniasis.

The other cytokines of IL-12 family, IL-23 and IL-27, can also stimulate a protective response against *L*. *infantum* [55,56]. Although some studies have associated IL-27with induction of IL-10 *in vivo* and suppression of immune response in VL [57], others have shown that it induces a STAT1/3- and NF-kappaB-dependent proinflammatory cytokine profile in human monocytes [49] and suppresses IL-10 production [50]. IL-1β has also been implicated in host resistance to *Leishmania* infection [2,5]. Signaling through IL-1R induces nitric oxide synthase (NOS2), which in turn mediates the production of NO [5]. Similar to our results, Ribbens et al showed that the addition of anti-CD40L to Th1/monocyte co-cultures can significantly reduce the production of IL-1β [58]. Confirming the effect of sCD40L present in sera on reduce the macrophage infection, we observed that recombinant CD40L (rCD40L) decrease the levels of infection and especially the number of intracellular parasites. Addition of rCD40L did not interfere with the phagocytosis, reinforcing that its action is in modulating the microbicidal response.

Further studies are required to more fully understand the effects of sCD40L in the presence of T cells or *in vivo*. However, the combination of our previous report [34], and the data presented here that demonstrate that engagement of, and signaling through, CD40 by sCD40L reduces parasite load *in vitro*, strongly indicate that sCD40L contributes to the control of infection.

In conclusion, our data suggest that sCD40L in the serum of *L*. *infantum*–infected individuals can enhance both the microbicidal response of infected macrophages and their production of inflamatory cytokines, each of which can promote killing of the parasites. Although datailed mechanistics studies are needed, this discovery suggests the potential use of sCD40L for future immunotherapy of human VL.
